# Bladder cancer and human papillomavirus association: a systematic review and meta-analysis

**DOI:** 10.1186/s13027-022-00415-5

**Published:** 2022-01-21

**Authors:** Alireza Khatami, Zahra Salavatiha, Mohammad Hossein Razizadeh

**Affiliations:** Department of Virology, Faculty of Medicine, Iran University of Medical Sciences, Tehran, Iran

**Keywords:** Human papillomavirus, HPV, Bladder cancer, Meta-analysis

## Abstract

**Background:**

The possible association of human papillomavirus (HPV) and bladder cancer has been controversial. Older findings suggest a significant association between the virus and bladder cancer. The aim of this study was to evaluate the data from the last ten years to estimate the prevalence of the virus in bladder cancer patients and to assess the association between the virus and cancer.

**Method:**

A search of major databases was conducted to retrieve published English language studies between January 2011 and March 2021. In the present study overall prevalence of the virus in bladder cancer patients was estimated along with the prevalence of subgroups. Also, the possible associations between the prevalence of the virus and bladder cancer and the possible impact of variables in the geographical area and the type of sample were measured by comprehensive meta-analysis software (V2.2, BIOSTAT).

**Results:**

Unlike previous studies, despite the relatively high prevalence of the virus [pooled prevalence: 14.3% (95% CI 8.9–22.2%)] no significant association was found between HPV and bladder cancer (OR 2.077, 95% CI 0.940–4.587). No significant association was found between geographical area (except Asia) and type of sample with bladder cancer.

**Conclusions:**

Given the significant prevalence, despite the insignificance of the association between virus and cancer, it seems that more studies with case–control design are needed to elucidate this association.

## Introduction

Bladder cancer is the tenth most common cancer globally, with 573,000 new cases and 213,000 deaths each year [1]. It is about four times more common in men than women, and it is the sixth most common cancer and the ninth leading cause of cancer death in men [2]. The well-known risk factors for bladder cancer include cigarette smoking, several occupations with exposures to aromatic amines (e.g. industrial dye manufacturing), the drug cyclophosphamide, and high use of the analgesic phenacetin [3]. There is an association between infection with parasitic agents (Schistosomiasis) and bacterial agents (Non-specific urinary tract infections Gonorrhea) with viral infections such as the Human papillomavirus (HPV), Human immunodeficiency virus, Bovine leukemia virus, BK virus, Herpes simplex virus [3–5]. Studies indicated that viruses cause 15–20% of all human cancers, of which about 10 percent are caused by HPV [6]. HPV is regarded as the most common viral sexually transmitted infection worldwide; more than 200 types of papillomavirus have been identified, which 14 types of them are regarded as the high-risk (HR) types, including 16, 18, 31, 33, 35, 39, 45, 51, 52, 56, 58, 59, 68, and 73 and have been observed in 90% of papillomavirus cancers [7–10]. HPVs are involved in the development of cervical, vagina, vulvar cancer in females, penile cancer in males, and anal cancers in both genders [11]. Due to the proximity of the genital tract and urinary tract, the risk of urinary tract infection with HPV is high [12]. Although various meta-analysis studies have been performed in different years on the possible role of HPV in the development of bladder cancer, the role of this virus in the development of bladder cancer is still controversial.

The present meta-analysis study reviewed articles over 10 years ago about the potential association of HPV in bladder cancer, besides the latest global prevalence of the virus in bladder cancer patients, also in this study, we discuss about the effects of various factors, including geographical distribution and type of sample used for virus detection.

## Methods

### Systematic search strategy

This study was conducted based on the PRISMA (Preferred Reporting Items for Systematic Reviews and Meta-Analyses) protocols. Main electronic databases include Medline (PubMed), Scopus, Web of Science, and Google Scholar were systematically searched by two investigators independently (A.K. and M.R.) for related articles published from January 2011 to March 2021. The used search keywords and terms were “HPV or human papillomavirus” AND “bladder cancer”. Additionally, the references of all original and review articles were hand-searched to finding other relevant studies.

### Including and excluding criteria

Eligible studies were analyzed by abstract and title. First, all the articles were imported into the Endnotes software, and in the next step, duplicated articles were then removed. The following inclusion criteria were applied for recruited publications in this study: (1) articles with case–control, cross-sectional, and cohort design; (2) published studies in the English language; (3) the papers with full-text, and (4) papers published from January 2011 to March 2021 with a digital object identifier (DOI); In addition, we also excluded studies with the following reasons: (1) Research on animals and in vitro; (2) all types of review papers, letters, comments, case reports, and case series.

### Data extraction

Data from all retrieved studies were extracted by two authors and double checked. If there were any discrepancies of opinion or disagreement between the two investigators, they would reach a consensus by consensus and discussion with the lead investigator (A.K.). We extracted the following variables from articles: first author’s last name, year of publication, country, total numbers of cases and controls, diagnosis methods, and type of sample.

### Quality assessment

We used the Joanna Briggs Institute (JBI) checklist for the quality assessment of the included articles [13], which contains ten questions with four answering options including, Yes, No, Unclear, and Not applicable; the overall score of each study can be a number between 1 and 10. Scores of 7–10 were considered high quality, and 4 to 6 were considered moderate quality for the studies. Therefore, we have decided to include and exclude the studies with 4 to 10 scores and ≤ 3 points, respectively.

### Data synthesis and statistical analysis

In the present systematic review and meta-analysis, we estimated the pooled odd ratio and prevalence of HPV in bladder cancer patients based on the random effect model (REM) with a 95% confidence interval (CI). The REM application lets for a distribution of true effect sizes between studies. Prevalence was also estimated based on subgroups of geographical area (continents), diagnostic method, cancer grade, sample type, virus genotype, HPV type (high risk and low risk), and gender of the subjects. In addition to the overall odd ratio, the geographic area and type of samples with bladder cancer association were calculated. The I2 statistic was performed to evaluate the studies' heterogeneity, and the publication bias was assessed by Egger’s regression test, and p-value < 0.05 was considered statistically significant; funnel plot represents the publication bias between studies. We applied the comprehensive meta-analysis software (version 2, Biostat, USA) for all analytical steps.

## Results

### Literature search

**Fig. 1:**
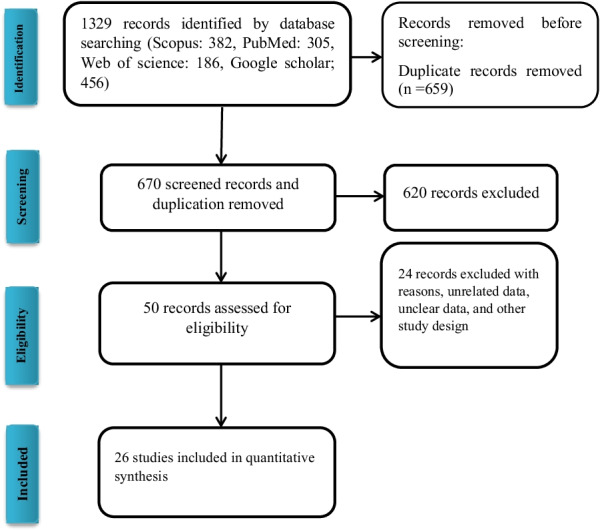
Flow diagram of the search strategy and studies selection process for studies in meta-analysis

**Fig. 2:**
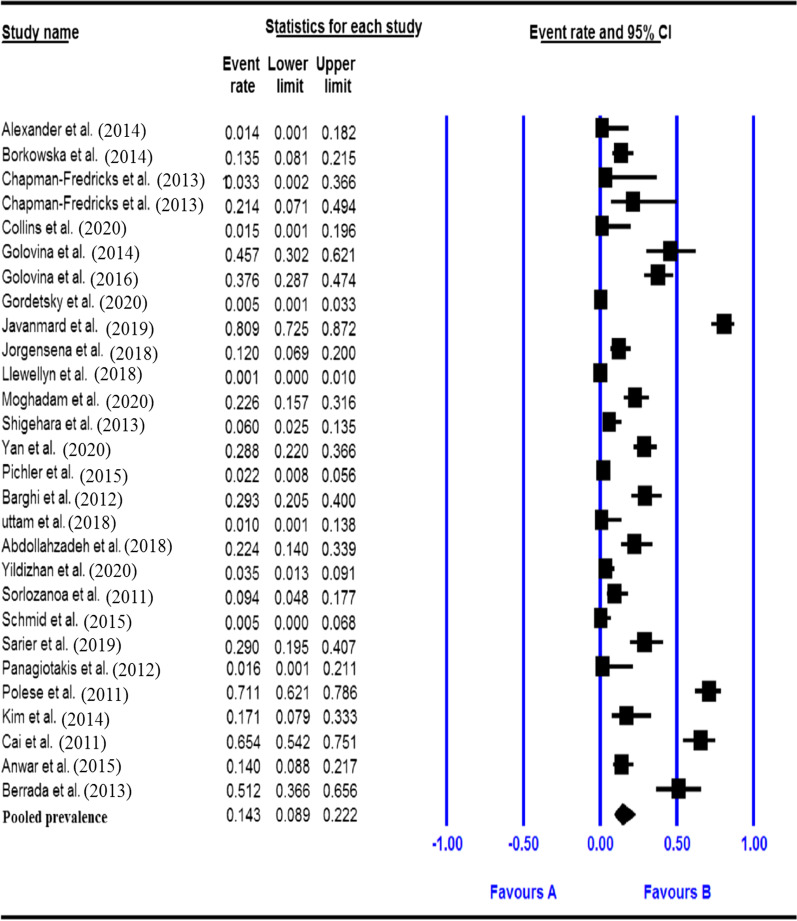
Forest plot of the pooled prevalence for HPV in bladder cancer

In the initial literature search strategy, a total of 1326 studies were identified from international electronic databases. In addition, 3 relevant papers were identified when searching for reference lists manually. Altogether, 659 duplicate studies were excluded, after the screening of 670 articles by title and abstract, which resulted in the removal of 620 articles. The remaining 51 articles were assessed for eligibility by the full-text review. After screening the full texts of the included articles, 24 studies were excluded based on reasons described in Fig. 1, and finally, 26 articles (27 datasets) were included in the meta-analysis (Fig. 2).

### Study characteristics

Twenty-seven datasets with a total of 2954 bladder cancer patients from eighteen countries were included in this study. The publication date of the studies was from January 2011 to March 2021. Twenty-seven records were selected for further analysis, of which 11 were from Asia, 1 study from Oceania, 4 studies from America, 3 studies from Europe, and 1 study from Africa. The characteristics of the selected studies of this analysis are summarized in Table 1.

**Table 1:** General characteristics of the included studies with case–control and cross-sectional design

First author	Study design	pub year	country	Continent	Method	Type of sample	Case	Positive (case)	Control	Positive (control)
Sorlozanoa et al. [14]	Case–control	2011	Spain	Europe	Nested PCR	Frozen	85	8	51	7
Polese et al. [15]	Case–control	2011	Italy	Europe	PCR	Frozen	114	81	140	108
Cai et al. [16]	Case–control	2011	Italy	Europe	PCR	Not indicated	78	51	59	21
Barghi et al. [17]	Cross-sectional	2012	Iran	Asia	PCR	FFPE	82	24		
Panagiotakis et al. [18]	Case–control	2012	Greece	Europe	PCR	Frozen	30	0	30	0
Chapman-Fredricks et al. [19]	Cross-sectional	2013	USA	America	ISH	FFPE	14	0		
Chapman-Fredricks et al. [19]	Cross-sectional	2013	USA	America	Hologic	FFPE	14	3		
Shigehara et al. [20]	Cross-sectional	2013	Japan	Asia	PCR	FFPE	84	5		
Berrada et al. [21]	Case–control	2013	Morocco	Africa	Nested PCR	Frozen	43	22	5	0
Alexander et al. [22]	Cross-sectional	2014	China	Asia	ISH	FFPE	36	0		
Borkowska et al. [23]	Cross-sectional	2014	Poland	Europe	Array	Not indicated	104	14		
Golovina et al. [24]	Cross-sectional	2014	Russia	Europe	PCR	Frozen	35	16		
Kim et al. [25]	Case–control	2014	Korea	Asia	Array	FFPE	35	6	12	1
Pichler et al. [26]	Cross-sectional	2015	Austria	Oceania	PCR	FFPE	186	4		
Schmid et al. [27]	Case–control	2015	Germany	Europe	PCR	Frozen	109	0	26	0
Anwar et al. [28]	Case–control	2015	Pakistan	Asia	PCR	FFPE	114	16	107	2
Golovina et al. [29]	Cross-sectional	2016	Russia	Europe	PCR	Frozen	101	38		
Jorgensena et al. [30]	Cross-sectional	2018	Denmark	Europe	INNO-LiPA	FFPE	100	12		
Llewellyn et al. [31]	Cross-sectional	2018	UK	Europe	PCR	Frozen	689	1		
Uttam et al. [32]	Cross-sectional	2018	India	Asia	PCR	FFPE/Frozen	50	0	10	0
Abdollahzadeh et al. [33]	Case–control	2018	Iran	Asia	IHC	FFPE	67	15	30	1
Javanmard et al. [34]	Cross-sectional	2019	Iran	Asia	PCR	FFPE	110	89		
Sarier et al. [35]	Case–control	2019	Turkey	Europe	PCR	Frozen	69	20	69	6
Collins et al. [36]	Cross-sectional	2020	USA	America	ISH	FFPE	33	0		
Gordetsky et al. [37]	Cross-sectional	2020	USA	America	ISH	FFPE	207	1		
Moghadam et al. [38]	Cross-sectional	2020	Iran	Asia	PCR	FFPE	106	24		
Yan et al. [39]	Cross-sectional	2020	China	Asia	NGS	Frozen	146	42		
Yildizhan et al. [40]	Case–control	2020	Turkey	Europe	PCR	FFPE	113	4	99	9

### Pooled prevalence of HPV in the bladder cancer patients

The total number of bladder cancer patients included in this meta-analysis was 2954 from adults based on the results of 27 datasets. The pooled prevalence of HPV infection among bladder cancer patients was 14.3% (95% CI 8.9–22.2%) based on a random-effects meta-analysis. In sub-group analysis by continent, the maximum and minimum prevalence of HPV infection among bladder cancer patients were found in Africa and Oceania, respectively (51.2, 95% CI 36.6–65.6% vs. 2.2, 95% CI 0.8–5.6%). Sub-group analysis based on HPV types showed that high-risk types had the highest prevalence (16.2%, 95% CI 9.8–25.5%), while low-risk types showed a lower prevalence (4.8%, 95% CI 2.2–10.2%). Details of the Pooled prevalence of HPV and bladder cancer risk for the subgroups are presented in Table 2.

**Table 2:** Overall prevalence and subgroup analysis results

Characteristics	Categories	No. of data sets	Pooled prevalence (%) (95% CI)	Heterogeneity		
				Q value	P-value	I^2^%
Overall	–	28	14.3 (8.9–22.2)	440.035	0.000	93.864
Continent	Africa	1	51.2 (36.6–65.6)	0.000	1.000	0.000
	America	5	3.1 (0.4–21.9)	12.795	0.005	76.553
	Asia	17	19.6 (10.1–34.7)	139.213	0.000	93.535
	Europe	3	14.9 (6.9–29.4)	223.190	0.000	95.071
	Oceania	8	2.2 (0.8–5.6)	0.000	1.000	0.000
Sample	FFPE	15	10.6 (51.1–21.0)	216.691	0.000	93.539
	Frozen	10	20.4 (10.5–35.9)	134.367	0.000	93.302
Method	Array	2	14.5 (9.5–21.4)	0.287	0.592	0.000
	ISH	4	1.1 (0.3–3.7)	1.367	0.713	0.000
	PCR	18	36.7 (33.4–40.1)	357.885	0.000	95.250
Grade	High	5	29.4 (19.0–42.6)	16.551	0.002	75.833
	Low	5	22.4 (13.1–35.5)	10.682	0.030	62.553
Genotype	HPV18	6	10.0 (4.9–19.2)	42.449	0.000	88.221
	HPV16	14	10.2 (5.4–18.5)	159.128	0.000	91.830
HPV type	High-risk	16	16.2 (9.8–25.5)	213.652	0.000	92.979
	Low-risk	5	4.8 (2.2–10.2)	12.515	0.014	68.039
Gender	Male	10	19.7 (12.9–28.8)	58.620	0.000	84.647
	Female	9	15.7 (8.1–28.3)	21.520	0.006	62.825

### The association of HPV with bladder cancer

**Fig. 3:**
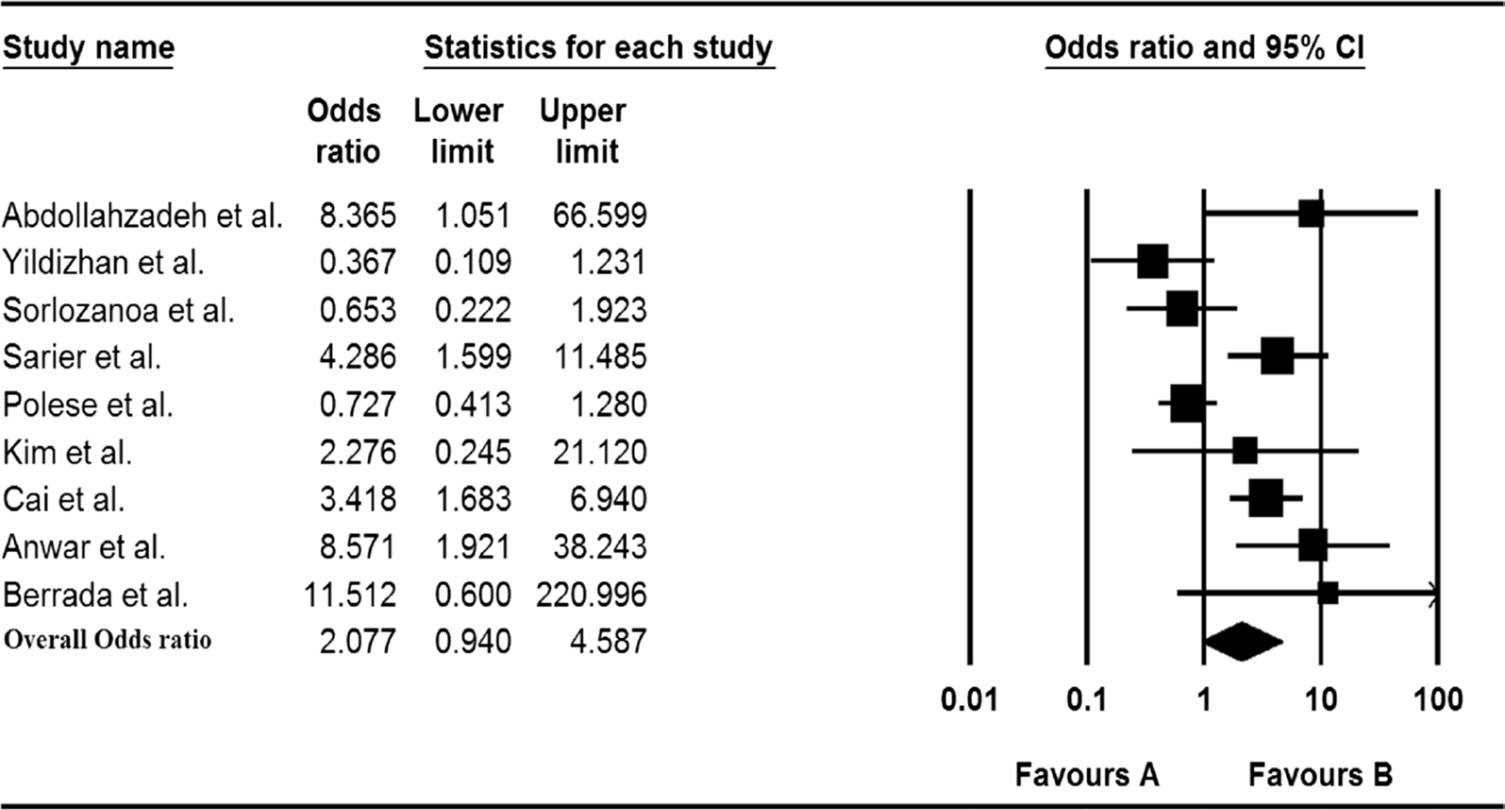
Forest plot of the overall odds ratio for association of HPV and bladder cancer

In 12 studies, the meta-analysis showed that HPV was not associated with bladder cancer, as shown in Fig. 3 [OR 2.077 (0.940–4.587)]. However, according to the subgroup analysis, the highest association between HPV and the risk of bladder cancer was in Asia (OR = 6.289; 95% CI 2.167–18.250). Details of the association between HPV and bladder cancer risk for the subgroups are presented in Table 3.

**Table 3:** Overall odd ratio and sub group analysis for case–control studies results

Characteristics	Categories	No. of data sets	Pooled OR (%) (95% CI)	Heterogeneity
				I^2^%
Overall	–	12	2.077 (0.940–4.587)	75.839
Continent	Africa	1	11.512 (0.600–220.996)	0.000
	Asia	4	6.289 (2.167–18.250)	0.000
	Europe	7	1.125 (0.503–3.113)	82.292
Sample	FFPE	4	2.584 (0.447–14.948)	76.856
	Frozen	6	1.559 (0.511–4.756)	76.355

### Publication bias

**Fig. 4:**
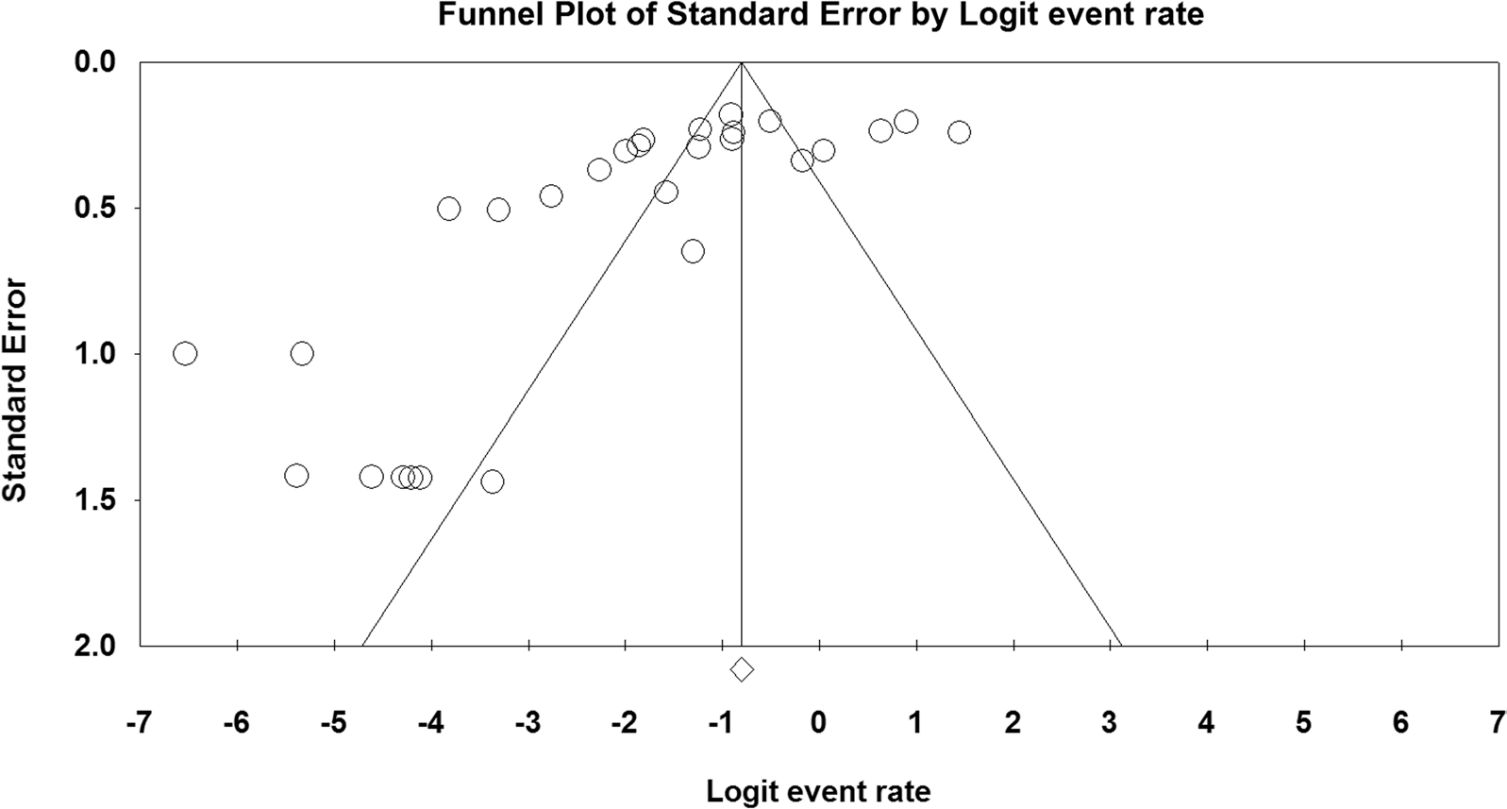
Funnel plot for publication bias assessment

Egger's regression test results for publication bias calculation showed the statistically significant (P < 0.000), this can be due to the low number of studies and/or studied subjects, the very high and/or low number of positive cases in some studies, the difference between diagnostic methods and the difference between operators skills (Fig. 4). The results of *I*^2^ statistics revealed significant heterogeneity among the included studies (df = 26, P < 0.000, I^2^ = 93.864%).

## Discussion

Bladder cancer is the tenth most common cancer in the world, with high morbidity and mortality rate. In addition to factors involved in cancer progressions such as gender, genetics, cigarette smoking, and exposure to polycyclic aromatic compounds, viral infections such as the human papilloma virus (HPV) have been introduced to be effective in the bladder cancer etiology and/or development [3]. The association between HPV and bladder cancer has been controversial, according to previous meta-analysis studies. However, with the relative increase in studies in this field and the comparatively suitable sample size for assessing the prevalence and association of HPV and bladder cancer, the present study addresses this relationship with more up-to-date information. Despite the latest meta-analysis results, our findings did not show a statistically significant association between HPV infection and bladder cancer [OR 2.077 (95% CI 0.940–4.587)]. The geographical spread of the virus and association with bladder cancer as well as the applied viral infections diagnostic samples and technique has always been interesting and obscure; in the last decade, the used diagnostic methods were mostly molecular, and the included studies for the present meta-analysis were also mostly molecular-based. The highest prevalence was according to the PCR method (36.7%, 95% CI 33.4–40.1%); this is probably due to the high number of studies performed with this technique and also the high sensitivity and specificity of this technique in viral diagnosis. Also, it seems that geographical distribution has an impact on the association between HPV and bladder cancer. Giving to the subgroup analysis by geographical area, unlike other areas, there was a significant relationship in Asia (OR 6.289, 95% CI 2.167–18.250); according to the study by Li et al., which is in line with our findings, this association could be related to genetics, ethnic, lifestyle, and even sexual behaviors as well as other unknown risk factors. However, it should be noted that the lack of association in other regions, especially Africa, is probably due to the small number of studies and the sample size. Up to now, the possible association of the virus in bladder cancer according to the diagnostic method has been discussed, but not about the sample taken for diagnosis. We analyzed the type of sample used to detect the virus in bladder cancer patients. The results showed no statistically significant relationship between the either frozen and/or FFPE sample type and detection of bladder cancer. Among the possible related factors, gender is controversial Most studies indicated that bladder cancer is more common in men than women [41, 42], but some studies showed that SCC of bladder cancer is more common in women than men [20, 30]. The results of our study showed a higher risk of bladder cancer in men than women (19.7%, 95% CI 12.9–28.8% versus 15.7% 95% CI 8.1–28.3%), which is in line with the study by Moghadam et al. [6].

Several studies have surveyed the prevalence of high-risk and low-risk types of virus and their involvement in the development of bladder cancer [17, 18, 43–45]. Based on our findings, the prevalence of high-risk types was higher (16.2%, 95% CI 9.8–25.5% versus 4.8%, 95% CI 2.2–10.2%), and in the case of high-risk genotypes, the prevalence of HPV 18 was not significantly different from that of HPV 16 (10.0%, 95% CI 4.9–19.2% versus 10.2%, 95% CI 5.4–18.5%).

However, we could not found any significant association between bladder cancer and HPV in our study; the results of our study are in line with various studies conducted by different groups in which no significant association was found between HPV infection and bladder cancer [14, 15, 18, 27, 28, 37, 45–52] In contrast, previous Meta-analyzes emphasized the significant association between virus and bladder cancer [44]. By analyzing newer studies, our results were inconsistent with previous Meta-analyzes; also, some studies have been failed to prove the association between HPV and SCC of bladder cancer and refuse the causative role of this virus [48, 53–55]. It also appears that HPV may be involved in the progression of different stages of bladder cancer due to the inactivation of tumor suppressors and a number of unknown mechanisms. The analysis results showed a high prevalence of the virus in the high grade of bladder cancer (Table 2).

The present study has faced limitations, such as the geographical limitations of the reports, so that reports were not available from some countries and continents according to the inclusion criteria. Other limitations included studies published in local languages.

## Conclusions

Despite the results of previous meta-analysis studies that reported the etiological role of HPV in bladder cancer, we conclude there is no significant association between HPV infection and bladder cancer. However, according to the analysis of studies of the last ten years, a relatively high prevalence of the virus was observed, which raises the possibility that with the increase of studies in this field, more comprehensive results will be obtained. Furthermore, the geographical area has a potential impact on the association between HPV and bladder cancer as a high prevalence of HPV infection was observed in Asia.

## Data Availability

All needed data are available in manuscript.
